# Two new *Erythrobasidium* species inhabiting the phyllosphere discovered in the Baotianman Nature Reserve in China

**DOI:** 10.3389/fmicb.2024.1287984

**Published:** 2024-02-06

**Authors:** Yun-Feng Lu, Chun-Yue Chai, Feng-Li Hui

**Affiliations:** ^1^School of Life Sciences and Agricultural Engineering, Nanyang Normal University, Nanyang, China; ^2^Research Center of Henan Provincial Agricultural Biomass Resource Engineering and Technology, Nanyang Normal University, Nanyang, China

**Keywords:** Basidiomycota, Erythrobasidiaceae, phylogenetic analysis, orange-red yeast, leaf

## Abstract

The genus *Erythrobasidium* is kind of species-scarce and undersampling basidiomycetes. Currently, only six species have been accepted into the genus and the diversity still remains incompletely understood. In this study, five *Erythrobasidium* strains were isolated in the surface of plant leaves collected from the Baotianman Nature Reserve, Henan Province, central China. Phylogenetic analyses of the small ribosomal subunit (SSU) rRNA gene, the internal transcribed spacer (ITS) region, the D1/D2 domain of the large subunit (LSU) rRNA gene, and the translation elongation factor 1-α (TEF1-α) gene coupled with morphological studies were employed to characterize and identify these isolates. As a result of these, two new species, namely *E. turpiniae* sp. nov. and *E. nanyangense* sp. nov., were delimited and proposed based on morphological and molecular evidence. A detailed description and illustration of both new species, as well as their differences with the close relatives in the genus are provided. An identification key for *Erythrobasidium* species is also provided. This study provides further insights into our understanding of *Erythrobasidium* species.

## Introduction

The genus *Erythrobasidium* is a type of phyllosphere-inhabiting Basidiomycota. It was defined by Hamamoto et al. (1988) to accommodate *Erythrobasidium hasegawae*, the teleomorphic state of *Rhodotorula hasegawae*. The original species name, *E. hasegawae*, was soon after changed to *E. hasegawianum* to fit standard naming conventions (Hamamoto et al., 1991; Hamamoto, 2011). *E. hasegawianum* was initially placed in the Agaricomycotina, but it was later reclassified as a member of the Pucciniomycotina based on phylogenetic analyses of the small subunit (SSU) rRNA gene (Hamamoto and Nakase, 2000), the D1/D2 domain of the large subunit (LSU) rRNA gene (Fell et al., 2000; Hamamoto, 2011), and a seven-gene dataset consisting of SSU, D1/D2 LSU, ITS, RPB1, RPB2, TEF1-α, and CYTB (Wang et al., 2015a). Wang et al. (2015b) revised the genus *Erythrobasidium* based on a multi-gene phylogeny, and transferred two *Sporobolomyces* species, *S. elongatum* and *S. yunnanensis*, to this genus as new combinations. Recently, three new species, *E. primogenitum*, *E. leptospermi*, and *E. proteacearum*, have been proposed by Tan et al. (2021) in Index Fungorum and by Tan and Shivas (2022) in Index of Australian Fungi based only on the D1/D2 and ITS sequences. Worryingly, the representative cultures of these three species are not currently available in a reference culture collection. The genus is presently classified in the family Erythrobasidiaceae within the order Erythrobasidiales, class Cystobasidiomycetes, Pucciniomycotina (Denchev, 2009; Wang et al., 2015b).

Except the type strain of *E. hasegawianum*, all strains of *Erythrobasidium* species have been reported to isolate from leaf surfaces (Shivas et al., 1983; Bai et al., 2001; Hamamoto, 2011; Hamamoto et al., 2011; Zang et al., 2018; Li et al., 2020; Tan et al., 2021; Tan and Shivas, 2022) and are considered important phyllosphere-inhabiting yeasts (Hamamoto, 2011; Hamamoto et al., 2011; Wang et al., 2015b). *E. hasegawianum*, the type species of the genus, is known from its sexual morph, which is characterized by unicellular basidia without mating and the lack of teliospores (Hamamoto, 2011). The other two validly known species, *E. elongatum* and *E. yunnanensis*, have been reported to have asexual morphs that reproduce by polar budding and the formation of ballistoconidia (Shivas et al., 1983; Bai et al., 2001; Hamamoto, 2011; Hamamoto et al., 2011). Physiologically, *Erythrobasidium* species can utilize various carbon and nitrogen sources, but not methanol or myo-inositol. From a phenotypic perspective, there are no distinctive phenotypic traits that can reliably delineate the genus *Erythrobasidium*. Therefore, molecular phylogenetic analysis coupled with morphological studies is recommended for identification of *Erythrobasidium* species (Wang et al., 2015b).

Species of the genus *Erythrobasidium* are best known for their orange-red colonies and have been studied for a variety of applications. For example, *Erythrobasidium* species have the ability to produce carotenoids such as beta-carotene. The carotenoid-producing capability of *Erythrobasidium* yeasts has been of interest to the field of biotechnology to develop commodities such as pigments (Mannazzu et al., 2015). *E. hasegawianum* is often fermented to produce linalool and ocimene, two common perfume components. As a natural bioflavoring producer, *E. hasegawianum* has great potential for applications in beverage industry.

The surface of plant leaves, normally referred to as the phylloplane, is known to be an important habitats for microorganisms. Various yeast species in the phyllosphere of different plants have been reported by several authors (de Azeredo et al., 1998; Zang et al., 2018; Srisuk et al., 2019; Into et al., 2020a,b; Li et al., 2020) but a few species of *Erythrobasidium* in this particular habitat have been uncovered. Surveying phylloplane can help us gain a better understanding of the diversity, distribution, and ecology of *Erythrobasidium* and lead to the discovery of new fungal species that may have valuable functions.

The Baotianman Nature Reserve located in Henan Province, central China, measures 4,285 ha. With a forest coverage rate of 98%, it is classified as World Biosphere Reserve by the United Nations Educational, Scientific and Cultural Organization (UNESCO). The reserve represents a virgin forest with more than 2,000 species of higher plants. The local climate consists of cold, dry winters and fresh, rainy summers—typical of a transitional zone from a northern subtropical zone to a warm temperate (Hu et al., 2022). These weather patterns make Baotianman an excellent location for studying fungal diversity. During the survey, a number of phyllosphere-inhabiting yeasts were obtained, and some of them have been described as new species, namely *B. ellipsoidea*, *B. foliicola*, and *B. pseudofoliicola*, in earlier paper (Chai et al., 2023). Among these associates, five additional yeast strains could not be ascribed to any validly known species. The aim of this study was to identify these yeasts as two new species of the genus *Erythrobasidium* based on multi-locus phylogenetic analyses of SSU, ITS, LSU, and TEF1-α sequence data and morphological observations.

## Materials and methods

### Sample collection and yeast isolation

Leaf samples collected from Baotianman Nature Reserve (33°30′44″N, 111°55′47″E) were stored in sterile flasks and transported to the laboratory within 24 h. Yeast strains were isolated from leaf surfaces by the improved ballistospore-fall method as described in previous paper (Nakase and Takashima, 1993). Briefly, the leaf was cut into small pieces that were attached with a thin layer of petroleum jelly to the inner lid of a Petri dish containing yeast extract-malt extract (YM) agar (0.3% yeast extract, 0.3% malt extract, 0.5% peptone, 1% glucose, and 2% agar) with added 0.01% chloramphenicol, to avoid bacterial growth. Plates were then incubated at 25 °C and monitored daily by eye for presence of colonies, which were selected and purified by streaking them on separate YM agar plates. After purification, yeast strains were suspended in YM broth supplemented with 20% (v/v) glycerol and stored at −80 °C. Cultures of all obtained isolates were preserved in Microbiology Lab, Nanyang Normal University, Henan, China. All isolates used in this study and their origins are presented in Table 1.

**TABLE 1:** Yeast strains and isolation sources investigated in this study.

Strain	Source	Location
***Erythrobasidium nanyangense***		
NYNU 208200^T^	Undetermined leaf	Baotianman Nature Reserve, Neixiang County, Henan Province, China
NYNU 20839	Undetermined leaf	Baotianman Nature Reserve, Neixiang County, Henan Province, China
NYNU 211295	Undetermined leaf	Baotianman Nature Reserve, Neixiang County, Henan Province, China
***Erythrobasidium turpiniae***		
NYNU 2110435^T^	Leaf of *Turpinia* sp.	Baotianman Nature Reserve, Neixiang County, Henan Province, China
NYNU 2110406	Undetermined leaf	Baotianman Nature Reserve, Neixiang County, Henan Province, China

### Morphological and physiological characterization

Morphological and physiological characteristics of yeast strains were defined according to methods established by Kurtzman et al. (2011). Colony characteristics were observed and recorded on YM agar after 2 weeks of incubation at 25 °C. To investigate mycelium formation, colonies were transferred to corn meal (CM) agar (2% cornmeal infusion and 2% agar) slide cultures and incubated at 25 °C for 2 weeks. Sexual tests were conducted for individual strains and strain pairs on potato dextrose agar (PDA) (20% potato infusion, 2% glucose, and 1.5% agar), CM agar, and yeast carbon base plus 0.01% ammonium sulfate (YCBS) agar for 2 months and observed at weekly intervals (Hamamoto, 2011; Li et al., 2020). The inverted-plate method (do Carmo-Sousa and Phaff, 1962) was used to observe the ballistoconidium-forming activity of all yeasts after 2 weeks of incubation on CM agar at 20 °C. Glucose fermentation was carried out in a liquid medium using Durham fermentation tubes. Carbon and nitrogen source assimilation tests were conducted in a liquid medium and starved inoculum was used for the nitrogen test (Kurtzman et al., 2011). Cycloheximide resistance was performed in a liquid medium, while urea hydrolysis was conducted on agar slants. Acid production and the diazonium blue B (DBB) reactions were investigated using petri dishes with a solid medium (Kurtzman et al., 2011). Growth at different temperatures (15, 20, 25, 30, 35, and 37 °C) was determined by the amount of cultivation on YM agar. All experiments were carried out with three replicates. Cell morphology was examined with LEICA DM2500 cameras (LECIA, Wetzlar, Germany) and use LASV4.13 software. At least 50 representative measurements were randomly selected and measured to calculate the average size of the budding cells. All novel taxonomic descriptions and proposed names were deposited in the MycoBank database.^1^

### DNA extraction, PCR amplification, and sequencing

The total genomic DNA was extracted from yeast strains using the Ezup Column Yeast Genomic DNA Purification Kit according to the manufacturer’s instructions (Sangon Biotech, China). Four nuclear loci, which include the SSU rRNA gene, the ITS region, the D1/D2 domain of the LSU rRNA gene, and TEF1-α gene were sequenced using NS1/NS8 (White et al., 1990), ITS1/ITS4 (White et al., 1990), NL1/NL4 (Kurtzman and Robnett, 1998), and EF1-526F/EF1-1567R (Rehner and Buckley, 2005) primers, respectively. The amplifications were performed in a 25 μL reaction-volume tube containing 9.5 μL of ddH_2_O, 12.5 μL of 2 × Taq PCR Master Mix with blue dye (Sangon Biotech, Shanghai, China), 1 μL of DNA template, and 1 μL of each primer. The following parameters were used to amplify the SSU, ITS, and D1/D2 regions: an initial denaturation step of 2 min at 95 °C, followed by 35 cycles of 30 s at 95 °C, 30 s at 51 °C, 40 s at 72 °C, and a final extension of 10 min at 72 °C (Toome et al., 2013). For TEF1-α, we used a touchdown PCR protocol as described (Wang et al., 2014). The PCR products were purified and sequenced at Sangon Biotech (shanghai) Co., Ltd (China) with the same primers. We determined the identity and accuracy of the newly-obtained sequences by comparing them to sequences in GenBank and assembled them using BioEdit 7.1.3.0 (Hall, 1999). All newly generated sequences were deposited in the GenBank database,^2^ and the accession numbers were listed in Table 2.

**TABLE 2:** DNA sequences used in molecular phylogenetic analysis.

Species name	Strain no.	GenBank accession no.			
		**SSU**	**ITS**	**LSU D1/D2**	**TEF1-α**
*Bannoa bischofiae*	JCM 10338^T^	AB035721	AB035721	NG_058609	AB127094
*Bannoa ellipsoidea*	JCM 35734^T^	OP221010	OM014197	OM014195	OP725922
*Bannoa foliicola*	CBS 16656^T^	OP218261	MW365541	MW365544	OP75051
*Bannoa guamensis*	CBS 16127^T^	MK254996	MK287350	MK255006	MK491345
*Bannoa hahajimensis*	JCM 10336^T^	AB035897	AB035897	NG_042311	KJ707750
*Bannoa ogasawarensis*	JCM 10330^T^	AB035717	AB035717	NG_058699	AB127095
*Bannoa rosea*	CBS 16128^T^	–	MK287351	MK255007	MK491346
*Bannoa syzygii*	JCM 10337^T^	AB035720	AB035720	NG_058700	AB127096
*Bannoa tropicalis*	CBS 16087^T^	MK255003	MK287360	MK255016	MK491353
*Bannoa pseudofoliicola*	JCM 35726^T^	OP221018	OM014200	OM014198	OP750518
*Cyrenella elegans*	CBS 274.82^T^	NG_061174	NR_145383	NG_058875	KJ707830
*Erythrobasidium elongatum*	CBS 8080^T^	NG_063449	NR_073306	NG_059254	AB127099
*Erythrobasidium hasegawianum*	JCM 1545^T^	D12803	NR_111008	AF131058	KJ707776
*Erythrobasidium leptospermi*	BRIP 66853^T^	–	NR_175759	NG_079571	–
***Erythrobasidium nanyangense***	**NYNU 208200** ^T^	**OP218268**	**MW362360**	**MW362359**	**OP313688**
***Erythrobasidium nanyangense***	**NYNU 20839**	**OR805565**	**OQ130167**	**OQ130166**	**OR785453**
***Erythrobasidium nanyangense***	**NYNU 211295**	**OR805564**	**OQ130172**	**OQ130170**	**OR785454**
*Erythrobasidium primogenitum*	BRIP 72389e^T^	–	**NR_182613**	**OP598058**	–
*Erythrobasidium proteacearum*	BRIP 66871^T^	–	**NR_175760**	**NG_079572**	–
***Erythrobasidium turpiniae***	**NYNU 2110435** ^T^	**OP218271**	**OM014199**	**OM014196**	**OR785452**
***Erythrobasidium turpiniae***	**NYNU 2110406**	**OR805578**	**OQ130168**	**OQ130169**	**OR785451**
*Erythrobasidium yunnanensis*	CBS 8906^T^	NG_063520	NR_155098	NG_059190	AB127100
*Hasegawazyma lactosa*	CBS 5826^T^	D45366	NR_073295	NG_057668	AB127098
*Naohidea sebacea*	CBS 8477^T^	KP216515	NR_121324	NG_042442	KJ707783

CBS, CBS-KNAW collections, Westerdijk Fungal Biodiversity Institute, Utrecht, Netherlands; JCM, Japan collection of microorganisms; BRIP, the plant pathology herbarium, Queensland, Australia; NYNU, Microbiology Lab, Nanyang Normal University, Henan, China; ^T^, type strain. Species obtained in this study are in bold.

### Phylogenetic analysis

A total of 21 taxa were included in the phylogenetic analyses in this study. Except for 20 sequences recognized in this study, the other sequences were obtained from previous studies (Wang et al., 2015b; Li et al., 2020) and GenBank (Table 2). *Naohidea sebacea* CBS 8477*^T^* was used as the outgroup. The phylogenetic relationships of the new *Erythrobasidium* species and their relatives were determined using a combined sequence dataset of four loci (SSU, ITS, LSU, and TEF1-α). Sequences of the individual loci were aligned with ClustalX v. 1.83 (Thompson et al., 1997) or MAFFT 7.110 (Katoh and Standley, 2013) using default settings. Phylosuit v. 1.2.2 (Zhang et al., 2020) was used to concatenate the aligned sequences of the different loci. Manual gap adjustments were performed to improve the alignment. Any ambiguously aligned regions were excluded before analysis.

Multi-locus phylogenetic analyses were carried out by using maximum likelihood (ML) and Bayesian inference (BI) methods. The ML was determined using 1,000 searches on RAxML v. 8.2.3 (Stamatakis, 2014). ML bootstrap values (BS) of the nodes were evaluated using 1,000 rapid bootstrap replicates under the GTRCAT model. For the BI approach, Modelfinder (Kalyaanamoorthy et al., 2017) was used to determine the appropriate substitution model that would best fit the DNA evolution for the combined dataset. MrBayes v. 3.2.7a (Ronquist et al., 2012) in the CIPRES Science Gateway version v. 3.3 was used to analyze the BI data. Best-fit evolution models were determined as GTR+I+G for the SSU, ITS, LSU, and TEF1-α partitions. Six simultaneous Markov chains were run for 50 million generations and trees were sampled every 1,000th generation. The first 25% of created sample trees were discarded as they represent the burn-in phase of analysis. The remaining trees were used to calculate Bayesian posterior probabilities (BPP) of the clades.

The resulting trees were viewed with FigTree v. 1.4.3 (Andrew, 2016) and processed with Adobe Illustrator CS5. Branches that received MLBS ≥ 50% and BPP ≥ 0.90 were considered significantly supported.

## Results

### Molecular phylogeny

**FIGURE 1:**
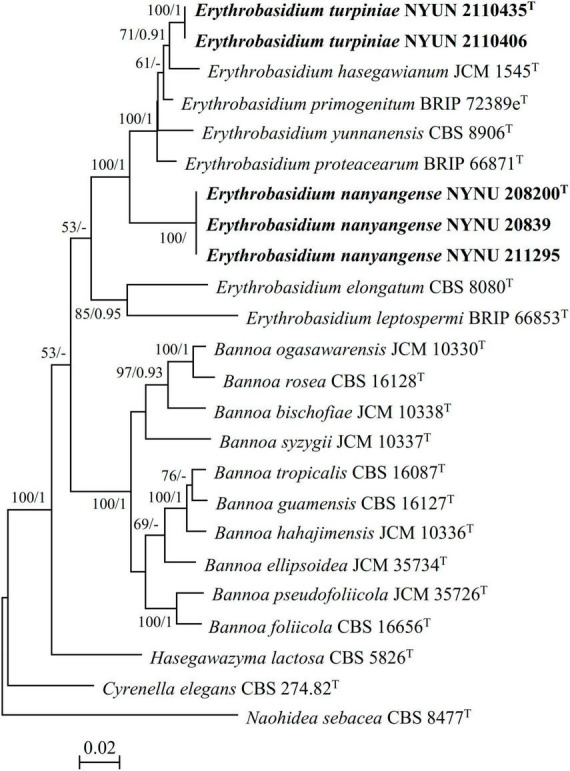
Maximum likelihood (ML) phylogram of the *Erythrobasidium* species based on combined SSU, ITS, LSU, and TEF1-α sequence data. *Naohidea sebacea* CBS 8477^T^ was used as the outgroup. Branches are labeled with MLBS ≥ 50% and BPP ≥ 0.90, respectively. New strains described in this study are shown in bold.

During this study, five strains of two new *Erythrobasidium* species were discovered in the Baotianman Nature Reserve. To reveal the phylogenetic position of the new species, we performed phylogenetic analyses with combined SSU, ITS, LSU, and TEF1-α sequence data. The sequence dataset consisted of 3,875 characters including gaps (SSU, 1,763 characters; ITS, 569 characters; LSU, 616 characters; TEF1-α, 927 characters). Of these characters, 2,528 were constant, 604 were variable but parsimony-uninformative, and 743 were parsimony-informative. The topology of ML tree is consistent with that of BI tree, agreeing with previous studies (Wang et al., 2015b; Li et al., 2020). Therefore, only the tree inferred from the ML analysis is provided in Figure 1 with MLBS (≥ 50%) and BPP (≥ 0.90) labeled on branches. In our phylogenetic tree, five newly isolated strains were formed into two well supported separate groups (100% MLBS/1 BPP) in the *Erythrobasidium* clade, and were clearly distinct from other species of *Erythrobasidium*.

Two strains NYNU 2110435 and NYNU 2110406 formed a well-supported clade that clustered with *E. hasegawianum* with moderate statistical support (71% MLBS/0.91 BPP) (Figure 1). The two strains of the NYUN 2110435 grope had identical sequences in both the D1/D2 domain and the ITS region, indicating they belong to the same species. This group differed from *E. hasegawianum* by four nt (∼0.7%) substitutions in the D1/D2 domain and by 9 nt (∼1.6) mismatches in the ITS region, respectively. In general, basidiomycetous yeast strains differing by two or more nucleotide substitutions in the D1/D2 domain or having nucleotide differences of 1-2% in the ITS region may represent different taxa (Fell et al., 2000; Vu et al., 2016). The differences in both the D1/D2 and ITS sequences have raised the possibility that the NYNU 2110435 group may represent a novel species distinct from *E. hasegawianum*. This hypothesis was supported by comparison of the partial TEF1-α gene sequences. While no difference among the partial TEF1-α gene sequences of the strains of the novel species, respectively, were detected; the novel species differed from *E. hasegawianum* by 40 nt (∼4.7 %) substitutions in this region. These findings confirm that the NYNU 2110435 group represents a novel species in the genus *Erythrobasidium*, for which the name *E. turpiniae* sp. nov. is proposed.

Three isolates NYNU 20839, NYNU 208200, and NYNU 211295 formed a distinct divergent lineage within *Erythrobasidium* (Figure 1). Three isolates of the NYNU 208200 grope shared 100% nucleotide identity based on the D1/D2 and ITS sequences, indicating that they are conspecific. BLASTn searches of the D1/D2 and ITS sequences indicated that this group was most closely related to *E. elongatum*, differing by 15 nt (∼2.6%) substitutions in the D1/D2 domain and 24 nt (∼4.3%) mismatches in the ITS region, respectively. Moreover, the partial TEF1-α gene sequences further confirm the novelty of this species, as they differed by 99 nt (∼11.6%) substitutions from *E. elongatum*. Hence, the NYNU 208200 group represents a novel *Erythrobasidium* species, for which the name *E. nanyangense* sp. nov. is proposed.

### Taxonomy

**FIGURE 2:**
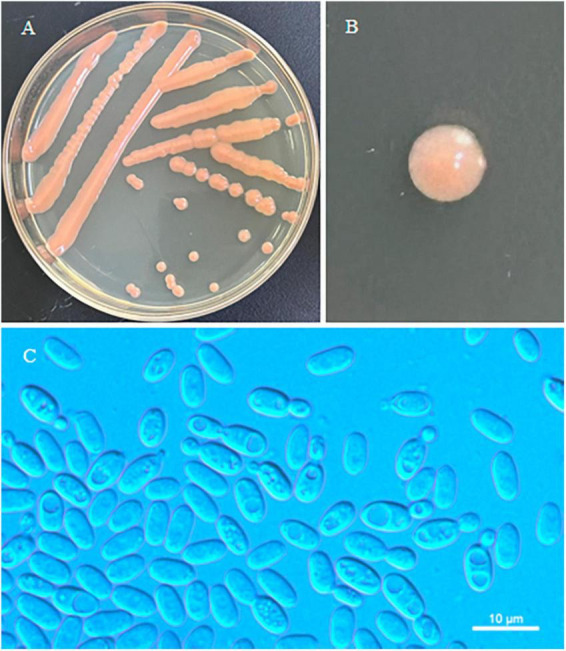
Morphological characteristics of *Erythrobasidium turpiniae* sp. nov (GDMCC 2.269, holotype). Culture **(A)**, single colony **(B)** and budding cells **(C)** on YM agar after growth for 7 d at 25 °C. Scale bars = 10 μm.

*Erythrobasidium turpiniae* C.Y. Chai & F.L. Hui, sp. nov., Figure 2.

**MycoBank: MB 847948**

Etymology: the specific epithet “*turpiniae*” refers to *Turpinia*, the plant genus, from which the type strain was isolated.

Typus: China, Henan Province, Neixiang County, Baotianman Nature Reserve, in phylloplane from leaf of *Turpinia* sp., October 2021, L. Zhang and H. Zhang, NYUN 2110435 (holotype GDMCC 2.269*^T^* preserved as a metabolically inactive state, culture ex-type JCM 35725).

Description: On YM agar, after 7 d at 25°C, the streak culture is orange, smooth, glistening and butyrous in texture. The margin is entire. On YM agar, after 7 d at 25 °C, cells are ellipsoidal and cylindrical, 2.8–4.9 μm × 5.3–7.7 μm and single, budding is polar. After 1 month at 25 °C, a ring and sediment are present. In Dalmau plate culture on corn meal agar, hyphae and pseudohyphae are not formed. Sexual structures are not observed for individual strains and strain pairs on PDA, CM agar and YCBS agar for 1 month. Ballistoconidia are not produced. Glucose fermentation is absent. Glucose, inulin, sucrose, galactose, lactose (weak), trehalose, maltose (weak), melezitose, cellobiose, salicin (weak), L-sorbose, L-rhamnose (weak), D-xylose, L-arabinose, D-arabinose (weak), 5-keto-D-gluconate (weak), glycerol, ribitol, D-mannitol, D-glucitol (weak), DL-lactate, succinate, citrate (weak), D-gluconate (weak), D-glucosamine, 2-keto-D-gluconate and D-glucuronate are assimilated as sole carbon sources. Raffinose, melibiose, methyl-α-D-glucoside, D-ribose, methanol, ethanol, erythritol, galactitol, myo-inositol and D-glucono-1,5-lactone are not assimilated. Nitrate, nitrite (weak), ethylamine (weak) and L-lysine are assimilated as sole nitrogen sources. Cadaverine is not assimilated. Maximum growth temperature is 30 °C. Growth in vitamin-free medium is negative. Starch-like substances are not produced. Urease activity is positive. Diazonium Blue B reaction is positive.

Additional strain examined: China, Henan Province, Neixiang County, Baotianman Nature Reserve, in phylloplane from undetermined leaf, October 2021, L. Zhang and H. zhang, 2110406.

GenBank accession numbers: holotype GDMCC 2.269*^T^* (SSU: OP221010, ITS: OM014199, D1/D2: OM014196, TEF1-α: OR785452); additional strain NYUN 2110406 (ITS: OQ130168, D1/D2: OQ130169, TEF1-α: OR785451).

Note: Physiologically, *E. turpiniae* sp. nov. can be differentiated from its closest relative *E. hasegawianum* (Hamamoto, 2011) by its ability to assimilate inulin, lactose, L-rhamnose, and D-gluconate, as well as its inability to assimilate D-ribose. In addition, *E. turpiniae* sp. nov. can grow at 30 °C while *E. hasegawianum* cannot (Table 3).

**TABLE 3:** Phenotypic characteristics that differ between the new species and closely related taxa.

Characteristics	*E. nanyangense*	E. hasegawianum*	E. yunnanensis*	*E. turpiniae*	E. elongatum*
**Carbon assimilation**					
Inulin	+	−	−	+	−
Galactose	+	s	w	+	−
Lactose	−	−	−	w	−
Methyl-α-D-glucoside	+	−	−	−	−
Cellobiose	−	s	s	+	+
L-Rhamnose	w	−	−	w	+
D-Ribose	w	d	−	−	−
Ethanol	−	−	l	−	−
Galactitol	+	−	−	−	−
D-Glucitol	+	+	−	w	+
Citrate	−	+	w	w	n
D-Gluconate	−	−	w	w	−
D-Glucosamine	−	s	n	+	−
2-Keto-D-gluconate	−	n	n	+	n
D-Glucuronate	+	+	−	+	−
D-Glucono-1,5-lactone	+	n	n	−	n
**Nitrogen assimilation**					
Nitrate	+	+	+	+	−
Nitrite	+	+	+	w	−
Ethylamine	w	+	−	w	−
L-Lysine	+	+	−	+	−
Cadaverine	w	−	−	−	−
Growth tests					
Growth at 30°C	+	−	−	+	−

+, positive reaction; −, negative reaction; d, delayed positive; l, latently positive; slowly positive; w, weakly positive; n, data not available. All data from this study, except * which were obtained from the original description (Hamamoto, 2011; Hamamoto et al., 2011).

**FIGURE 3:**
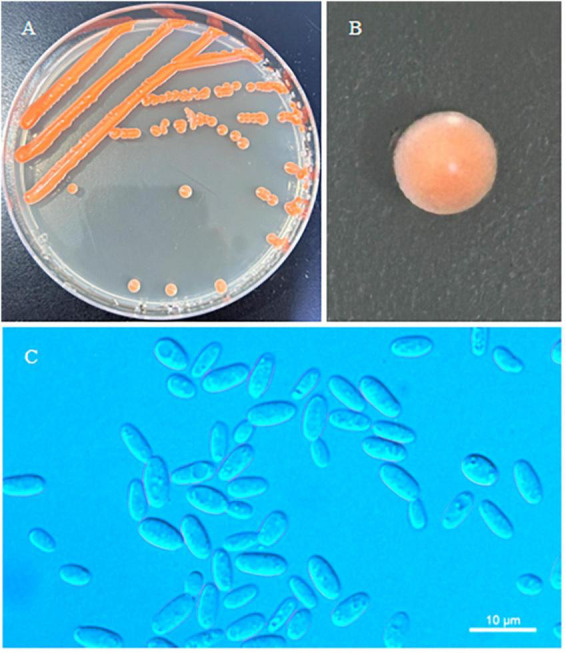
Morphological characteristics of *Erythrobasidium nanyangense* sp. nov (CICC 33505, holotype). Culture **(A)**, single colony **(B)** and budding cells **(C)** on YM agar after growth for 7 d at 25 °C. Scale bars = 10 μm.

*Erythrobasidium nanyangense* C.Y. Chai & F.L. Hui, sp. nov., Figure 3.

**MycoBank: MB 847949**

Etymology: the specific epithet “*nanyangense*” refers to the geographic origin of the type strain, Nanyang city, Henan Province.

Typus: China, Henan Province, Neixiang County, Baotianman Nature Reserve, in phylloplane from undetermined leaf, July 2016, L. Zhang and H. zhang, NYNU 208200 (holotype CICC 33505*^T^* preserved as a metabolically inactive state, culture ex-type CBS 16661).

Description: On YM agar, after 7 d at 25 °C, the streak culture is orange–red, smooth, glistening and butyrous in texture. The margin is entire. In YM broth, after 7 d at 25 °C, cells are ovoid or cylindrical, 2.5–3.7 μm × 4.8–7.8 μm and single, budding is polar. After 1 month at 25 °C, a ring and sediment are present. In Dalmau plate culture on corn meal agar, hyphae and pseudohyphae are not formed. Sexual structures are not observed for individual strains and strain pairs on PDA, CM agar and YCBS agar for 1 month. Ballistoconidia are not produced. Glucose fermentation is absent. Glucose, inulin, sucrose, galactose, trehalose, maltose, melezitose, methyl-α-D-glucoside, salicin, L-sorbose (weak), L-rhamnose (weak), D-xylose, L-arabinose, D-arabinose, 5-keto-D-gluconate, D-ribose (weak), glycerol, ribitol, galactitol, D-mannitol, D-glucitol, DL-lactate (weak), succinate, D-glucuronate and D-glucono-1,5-lactone are assimilated as sole carbon sources. Raffinose, melibiose, lactose, cellobiose, methanol, ethanol, erythritol, myo-inositol, citrate, D-gluconate, D-glucosamine and 2-keto-D-gluconate are not assimilated. Nitrate, nitrite, ethylamine (weak), L-lysine and cadaverine (weak) are assimilated as sole nitrogen sources. Maximum growth temperature is 30 °C. Growth in vitamin-free medium is negative. Starch-like substances are not produced. Urease activity is positive. Diazonium Blue B reaction is positive.

Additional strain examined: China, Henan Province, Neixiang County, Baotianman Nature Reserve, in phylloplane from Undetermined leaf, October 2021, L. Zhang and H. zhang, NYNU 20839 and NYUN 211295.

GenBank accession numbers: holotype CICC 33505*^T^* (ITS: MW362360, D1/D2: MW362359, TEF1-α: OP313688); additional strains NYNU 20839 (ITS: OQ130167, D1/D2: OQ130166, TEF1-α: OR785453) and NYUN 211295 (ITS: OQ130172, D1/D2: OQ130170, TEF1-α: OR785454).

Note: Physiologically, *E. nanyangense* sp. nov. can be differentiated from its closest relative, *E. elongatum* (Hamamoto et al., 2011), by its ability to assimilate inulin, galactose, methyl-α-D-glucoside, D-ribose, galactitol, D-glucuronate, nitrate, nitrite, ethylamine, L-lysine, and cadaverine, as well as its inability to assimilate cellobiose. In addition, *E. nanyangense* sp. nov. can grow at 30 °C while *E. elongatum* cannot (Table 3).

### Key to species of *Erythrobasidium*

The four species now recognized in *Erythrobasidium* can be differentiated as indicated in the following key:

1. a. Methyl-α-D-glucoside is assimilated . . . . . . . . . . . . . . . . . . . . . . . *E. nanyangense*
b. Methyl-α-D-glucoside is not assimilated . . . . . . . . . . . . . . . . . . . . . . . . . . . . . . . . . .2
2. (1) a. Inulin is assimilated . . . . . . . . . . . . . . . . . . . . . . . . . . . . . . . . . . . . . . *E. turpiniae*
b. Inulin is not assimilated . . . . . . . . . . . . . . . . . . . . . . . . . . . . . . . . . . . . . . . . . . .3
3. (2) a. D-Glucuronate is assimilated . . . . . . . . . . . . . . . . . . . . . . . . . . . .*E. hasegawianum*
b. D-Glucuronate is not assimilated . . . . . . . . . . . . . . . . . . . . . . . . . . . . . . . . . . . . . . 4
4. (3) a. D-Glucitol is assimilated . . . . . . . . . . . . . . . . . . . . . . . . . . . . . . . . .*E. elongatum*
b. D-Glucitol is not assimilated . . . . . . . . . . . . . . . . . . . . . . . . . . . . . . . .*E. yunnanensis*

## Discussion

Traditional methods of classification for *Erythrobasidium* species are based primarily on phenotypical features, such as colony morphology, cell shape, basidia formation, and details of physiological and biochemical characteristics etc. (Hamamoto, 2011). The classification based on these phenotypical features, however, was in many cases not consistent with the results obtained from phylogenetic analyses. For example, *E. elongatum* and *E. yunnanensis*, originally classified in the *Sporobolomyces*, are nested within the teleomorphic genus *Erythrobasidium* based on phylogenetic analyses (Bai et al., 2001; Hamamoto, 2011; Hamamoto et al., 2011; Wang et al., 2015a). As a result, these two species were then reassigned to the genus *Erythrobasidium*, according to the International Code of Nomenclature for Algae, Fungi, and Plants (McNeill et al., 2012). Therefore, a combination of phenotypical characteristics and phylogenetic analysis has been adopted as the standard method for concretely identifying *Erythrobasidium* species (Wang et al., 2015b).

In this study, we introduce *E. turpiniae* sp. nov. and *E. nanyangense* sp. nov as two new species of *Erythrobasidium*, and describe them in asexual morphs based on molecular analyses and morphological features. We found that *E. turpiniae* sp. nov. formed a basal clade related to *E. hasegawianum* (Figure 1). *E. nanyangense* sp. nov. clustered together with a separate clade within *Erythrobasidium* (Figure 1). We compared the sequences of the D1/D2 domain, ITS region, and TEF1-α gene of two new species with their closely related species. The differences in these regions were great enough to separate new *Erythrobasidium* strains into two species. The species shared high similarity in colony morphology, color, and individual cell shape, and they clearly differed from the closest known species in physiological and biochemical characteristics (Table 3). The combination of the morphological characteristics and molecular analyses recorded in our study strongly supports the recognition of two new *Erythrobasidium* species.

Members of the genus *Erythrobasidium* have not yet been sufficiently studied and the species diversity has long been underestimated. Up to now, only eight *Erythrobasidium* species, including *E. turpiniae* sp. nov. and *E. nanyangense* sp. nov. described in the present study, were found in nature. *E. hasegawianum* was the most widely distributed, occurring in China, USA, and South Africa (Hamamoto, 2011; Vu et al., 2016; Zang et al., 2018; Li et al., 2020). *E. elongatum*, *E. leptospermi*, *E. primogenitum*, *E. proteacearum*, *E. nanyangense* sp. nov., *E. turpiniae* sp. nov., and *E. yunnanensis* were scarce, occurring only in Australia or China (Bai et al., 2001; Hamamoto et al., 2011; Tan et al., 2021; Tan and Shivas, 2022). However, some unpublished strains of *Erythrobasidium* have also been isolated in different parts of the world; for example, *Erythrobasidium* sp. GY113362PS (LC272891) and *Erythrobasidium* sp. GY1131127PS (LC272890) have been obtained from Korea, *Erythrobasidium* sp. OTU655 (MK018684) and *Erythrobasidium* sp. LM681 (EF060964) from USA, *Erythrobasidiales* sp. DBP-2011 (KM527115) from Italy, and *Erythrobasidium* sp. UFMG-ABT330 (KM527115) and *Erythrobasidium* sp. BRT565 (OR430047) from Brazil. In addition, several environmental sequences of *Erythrobasidium* have also been reported from Brazil, from Finland (Pitkäranta et al., 2008), and from Germany (Renker et al., 2005; Neubert et al., 2006). Taken together, these suggests this genus could be broadly distributed and further large-scale studies are needed to explore the diversity and distribution of *Erythrobasidium* species worldwide.

The phylloplane is an important habitat for yeasts and these yeasts can alter nutrient availability for other microorganisms, antagonize pathogens, and stimulate plant defenses (Into et al., 2020a,b). As yeasts play crucial roles in nutrient cycling and symbiotic relationships with plants, discovering the diversity of yeast taxa helps in the functioning of ecosystems. Besides, protected areas like the Baotianman Nature Reserve are usually undisturbed, and conducting research in these areas has a high potential for discovering novel and endemic yeast species, consequently contributing to the expansion of yeast diversity.

## Conclusion

Although *Erythrobasidium* is widely distributed in the world, the diversity of this genus has not been completely resolved. In this work, five phyllosphere-inhabiting yeast strains were identified as two new species, *E. turpiniae* sp. nov. and *E. nanyangense* sp. nov., based on morphological and molecular phylogenetic analysis, which provides us with further understanding of this genus diversity in China. In the future, we firmly believe that more and more species of the genus will be isolated from more plants around the world.

## Data availability statement

The datasets presented in this study can be found in online repositories. The names of the repository/repositories and accession number(s) can be found in this article/supplementary material.

## Author contributions

Y-FL: Investigation, Writing – original draft. C-YC: Investigation, Writing – review & editing. F-LH: Writing – review & editing.

## Footnotes

1
http://www.mycobank.org

2
https://www.ncbi.nlm.nih.gov/genbank/
